# Targeting Nutrient Dependency in Cancer Treatment

**DOI:** 10.3389/fonc.2022.820173

**Published:** 2022-02-01

**Authors:** Kexin Fan, Zhan Liu, Min Gao, Kangsheng Tu, Qiuran Xu, Yilei Zhang

**Affiliations:** ^1^ The Institute of Molecular and Translational Medicine, Department of Biochemistry and Molecular Biology, School of Basic Medical Sciences, Xi’an Jiaotong University Health Science Center, Xi’an, China; ^2^ Department of Biochemistry and Molecular Biology, School of Basic Medical Sciences, Xinjiang Second Medical College, Karamay, China; ^3^ Department of Hepatobiliary Surgery, the First Affiliated Hospital of Xi’an Jiaotong University, Xi’an, China; ^4^ The Key Laboratory of Tumor Molecular Diagnosis and Individualized Medicine of Zhejiang Province, Zhejiang Provincial People’s Hospital, Affiliated People’s Hospital, Hangzhou Medical College, Hangzhou, China; ^5^ Research Center of Diagnosis and Treatment Technology for Hepatocellular Carcinoma of Zhejiang Province, Hangzhou, China

**Keywords:** nutrient, dependency, cancer, therapy, metabolism

## Abstract

Metabolic reprogramming is one of the hallmarks of tumor. Growing evidence suggests metabolic changes that support oncogenic progression may cause selective vulnerabilities that can be exploited for cancer treatment. Increasing demands for certain nutrients under genetic determination or environmental challenge enhance dependency of tumor cells on specific nutrient, which could be therapeutically developed through targeting such nutrient dependency. Various nutrients including several amino acids and glucose have been found to induce dependency in genetic alteration- or context-dependent manners. In this review, we discuss the extensively studied nutrient dependency and the biological mechanisms behind such vulnerabilities. Besides, existing applications and strategies to target nutrient dependency in different cancer types, accompanied with remaining challenges to further exploit these metabolic vulnerabilities to improve cancer therapies, are reviewed.

Tumor metabolism has emerged to be an attractive topic in the field for many years, considering that substantial evidence and insights are presented by a huge number of great studies. Reprogramming and rewiring of metabolic pathways to either adapt to stressful environments or to meet their own dramatic demands during tumor expansion is widely recognized and plays an indispensable role in cancer development (1–3). Extracellular nutrients, including amino acids, glucose and lipids, are major resources to drive the metabolic engine within tumor cells. Under certain circumstances, like genetic mutations, alterations of metabolic gene expression and limitations of nutrient supply in the tumor environment, tumor cells exhibit relatively high addition to one particular nutrient, which creates nutrient dependency that could be therapeutically targeted in cancer treatment (4–6). Thus, restricting nutrient availability by various means such as dietary approaches and amino acids degrading enzymes causes growth arrest, cell death and, partly, if not all, tumor suppression, which acts as an anti-cancer strategy and is definitely worth further study (7–9). In addition, nutrient availability also affects numerous cell types within tumor microenvironment and malignant cells undergo many challenges as well as compensations from other types of cells in the context, which is assumed as heterocellular metabolic interactions that impede our precise understanding of tumor metabolism (10).

Interfering with nutrient availability can be secretively lethal to tumor cells, which serves as a cancer-specific Achilles’ heel. To date, selective dependencies of tumor cells on amino acids such as asparagine, arginine, methionine, glutamine and cysteine, or the major energy source glucose have been wildly documented, although the underlying mechanisms vary and are highly context dependent (9). How do genetic mutations influence metabolic fluxes? How does metabolic reprogramming control nutrient dependency? What vulnerabilities do these alterations expose and can they be therapeutically targeted? In this review, we focus on the regulation of metabolism in tumor cells and discuss the key concepts for targeting nutrient dependency developed in the past few years as well as the most recent progresses on this emerging topic.

## Nutrients Causing Dependency of Tumor Cells

**Figure 1:**
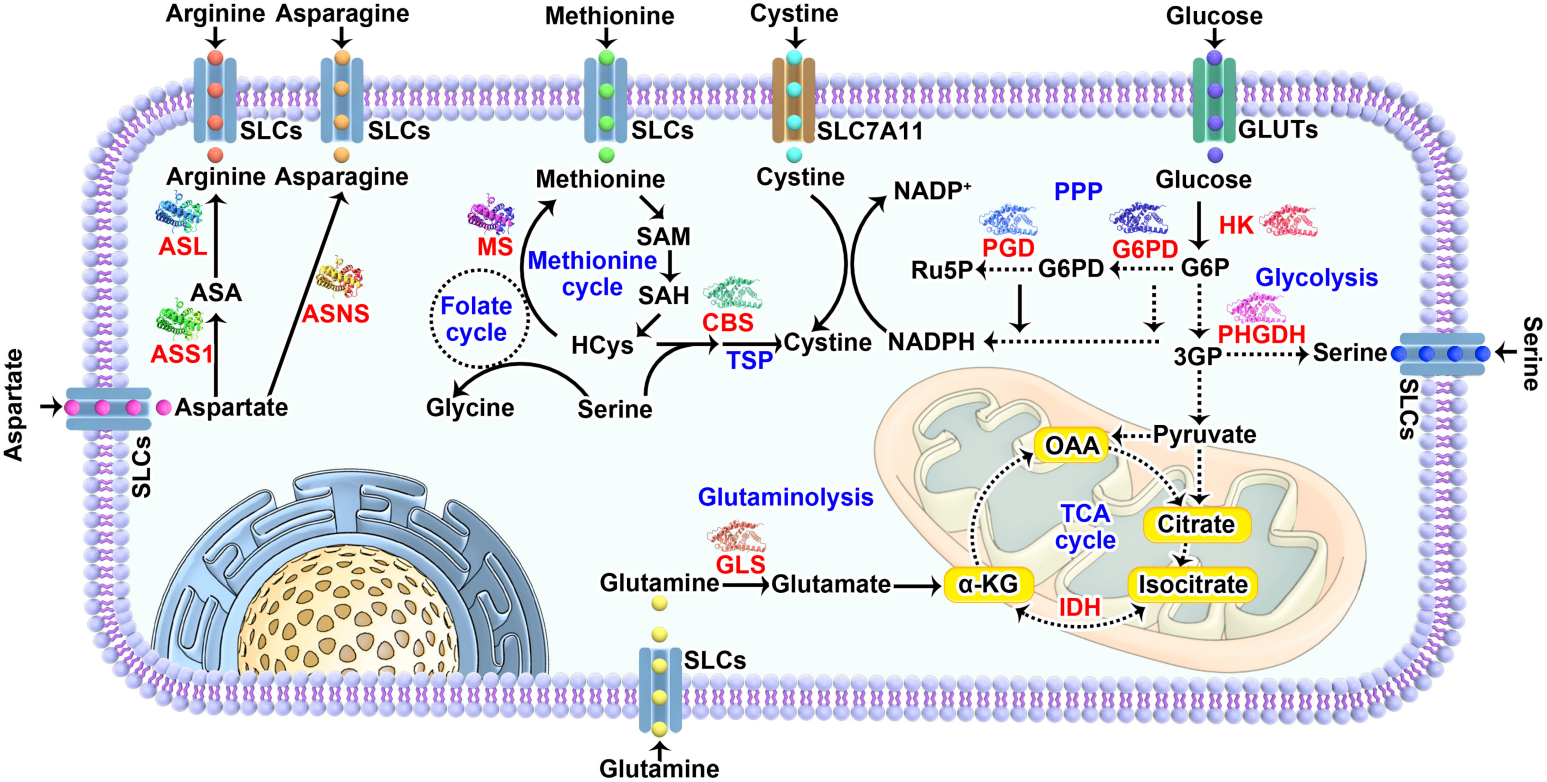
Nutrients that frequently causing dependency in tumor cells. The major pathways and key enzymes (labeled in red) involved that modulating the dependency of discussed nutrients (labeled in purple) are summarized in the diagram. SLCs, solute carrier-type transporters; GLUTs, glucose transporters; ASS1, argininosuccinate Synthase 1; ASL, argininosuccinate lyase; ASNS, asparagine synthetase; MS, methionine synthase; CBS, cystathionine beta-synthase; PGD, phosphogluconate dehydrogenase; G6PD, glucose-6-phosphate dehydrogenase; HK, hexokinase; PHGDH, phosphoglycerate dehydrogenase; IDH, isocitrate dehydrogenase; GLS, glutaminase; TSP, transsulfuration pathway; PPP, pentose phosphate pathway; TCA cycle, the tricarboxylic acid cycle; HCys, homocysteine; NADPH, reduced nicotinamide adenine dinucleotide phosphate; G6P, glucose 6-phosphate; 3PG, 3-phosphoglycerate; 6PD, 6-phosphogluconate; Ru5P, ribulose 5-phosphate; OAA, oxaloacetate; α-KG, α-ketoglutarate.

Nutrients like amino acids, glucose, lipids, vitamins, inorganic salt and trace elements are required for the growth of all types of cells and maintaining a steady state in response to environmental challenges. Therefore, it’s rarely impossible to specifically target nutrient availability in tumor cells while leaving normal cells untouched. Due to cell-autonomous metabolic reprogramming, tumor cells are relatively more dependent on one or more nutrients to support their core functions: biomacromolecules synthesis, energy formation, redox control and stress response (3). So far, the roles of several amino acids and glucose in nutrient dependency are extensively studied, which will be further discussed in the following section (**Figure 1**). In addition, we will also briefly discuss nutrient dependency caused by lipids and vitamins to spark any ideas about targeting their metabolism in cancer treatment.

### Arginine

Arginine is a conditionally essential amino acid or semi-essential amino acid, which means it occasionally needs supplement from dietary intake (11). *In vivo*, Arginine is synthesized from aspartate or citrulline through argininosuccinate synthetase (ASS1) and argininosuccinate lyase (ASL) (12), which act as key regulators in determining the arginine-dependency of tumor cells. Due to deregulation of ASS1 or ASL (such as loss of ASS1), tumor cells have much a higher demand on extracellular arginine than their normal counter parts, leading to arginine auxotrophy (13–16). Consequently, depleting arginine through arginase (ARGase, converting arginine into ornithine and urea) or arginine deiminase (ADI, converting arginine into citrulline and NH_3_) shows great potential in triggering cell death or reducing tumor growth in various cancer types including non-small cell lung cancer, glioblastoma, bladder cancer, pancreatic cancer, liver cancer, leukemia and melanoma (11, 16–21). However, the anti-cancer effects haven’t reached our expectation when using ARGase treatment for cancer patients, possibly due to limited efficacy of ARGase *in vivo* or compensatory arginine supply from amino acids-related salvage pathways (9). Alternatively, ADI treatment-mediated arginine deprivation exhibits acceptable tolerance and has been brought into clinical trials in several types of cancers (22, 23), encouraging persistent dedication to deeply exploring the applications of targeting arginine dependency in cancer treatment. Of note, treatment with pegzilarginase, an engineered human ARGase with superior stability and catabolic activity, indirectly augments immune response and dramatically improves M1-like antitumor macrophages, eventually synergizing with anti-PD-L1 treatment to suppress tumor (24). This finding provides new insights for the clinical evaluation of targeting arginine dependency in conjunction with immune checkpoint blockade therapy.

### Asparagine

Asparagine is a non-essential amino acid which could be synthesized through asparagine synthetase (ASNS)-from aspartate in an ATP-dependent manner (25). However, the demonstration of its metabolic requirement for tumor cell growth makes it an ideal target in cancer treatment (26). Indeed, enzymatic degradation of asparagine by L-asparaginase (ASNase, converting asparagine into aspartic acid and ammonia) treatment exhibits efficient tumor regression in acute lymphoblastic leukemia (ALL) and is the most successful and best documented nutrient dependency-targeting therapy in anti-cancer treatments (27). The efficacy of ASNase treatment seems to be dependent on ASNS expression within tumor cells (28, 29), though the detailed reasons for asparagine dependency of leukemias remain further investigations. Therefore, ASNS might be a suitable biomarker considering its low expression at least in part benefits ASNase therapy in ALL treatment. If so, a better understanding of the mechanistic regulation of ASNS in ALLs would contribute to deep exploration on asparagine-limitation induced anti-cancer effects. To date, epigenetic regulations of ASNS like DNA methylation, histone methylation and acetylation have been proved to control ASNS expression in human leukemic cell lines as well as leukemia patients (30, 31). Thus, it prompts a logical theory of combined treatment with ASNase, which depletes asparagine in the extracellular context, and DNA demethylase or histone acetyltransferase inhibitors, which bring down the expression level of ASNS inside cells, and ideally this should be more relevant to ASNase-resistant ALLs, yet to be demonstrated in the future study. An encouraging study shows that compound APR-246 which directly targets ASNS induces synergistic growth suppression when combined with ASNase treatment in ALL cells (32). Like all other chemical drugs, ASNase is not absolutely specific and able to degrade glutamine due to its partial glutaminase activity, which causes cytotoxicity and side-effects during treatment (33, 34). Presumably, the anti-cancer effects of dual-enzyme activities of ASNase could be explained by the co-dependency of glutamine/glutamate and asparagine/aspartate observed in several studies (35, 36). In addition to glutamine, thiamine-restriction also sensitizes a subset of leukemia cells to ASNase treatment (37). These findings call for more comprehensive studies on therapeutic response to ASNase.

### Glutamine

Glutamine is a non-essential amino acid that can be synthesized from glucose but plays an essential role in maintaining the growth of some cancer cells *in vitro*, which is known as “glutamine addiction” (38, 39). Glutamine has multiple independent functions: serving as the key nitrogen donor for protein and nucleotide synthesis, supporting the uptake of certain essential amino acids and maintaining tricarboxylic acid (TCA) cycle as well as redox balance through glutaminolysis (40–42). Therefore, targeting glutamine dependency has been reported in different contexts involving its intrinsic functions as mentioned above. For instance, pancreatic ductal carcinoma (PDAC) cells with oncogenic KRAS are particularly dependent on glutamine metabolism-mediated NADPH generation, which potentially maintain cellular redox state (43). Similarly, colorectal cancer with oncogenic PIK3CA mutations exhibits strong dependency on glutamine due to up-regulated expression of glutamate pyruvate transaminase 2 (GPT2) (44). In addition, other key regulators like glutamine synthetase (GS), LKB1 (liver kinase B1), KEAP1, c-Myc and FLT3 are also found to control glutamine dependency in various contexts (45–49). Therefore, the application of glutamine targeted therapies is routinely exploited in different cancer types. Strategies of targeting glutaminase (which generates glutamate from glutamine) or glutaminolysis pathway proteins are developed to suppress tumor growth in leukemia, liver and pancreatic cancers, all of which showed therapeutical efficacy at least in preclinical studies (50–55). Considering the interplay between glutamine and other nutrients, targeting one nutrient might lead to co-dependency on glutamine. Metformin treatment-induced glucose oxidation inhibition can cause dependency on reductive glutamine metabolism in prostate cancer cells (56). Moreover, tumor cells are able to survive and adapt to the poor nutrient environment through metabolic rewiring of mTORC1 activity, which stabilizes GS to support tumor growth under nutrient-deprived microenvironments (57). These findings provide potential additional therapeutic targets such as glucose transporters and mTORC1, inhibition of which could be synergized with glutamine dependency targeted therapies, yet to be demonstrated in the future.

### Methionine

Methionine is one of the major essential amino acids playing vital roles in protein synthesis, generation of S-adenosylmethionine (SAM, the sole methyl donor for methylation of DNA, histones and proteins), redox homeostasis (contribute to cysteine and glutathione synthesis) and nucleotide biosynthesis (polyamines) (58). As early as the 1970s, malignant and transformed cells, unlike normal cells, have been found not growing or surviving in methionine-deficient and homocysteine-supplemented media, which is referred to as methionine dependency of cancer or the Hoffman effect (59). The high reliability of cells on methionine have been demonstrated in multiple types of cancers including breast, pancreatic, colon, prostate, lung, kidney cancer and leukemia (58, 60). The mechanisms causing methionine dependency in tumor cells have been discussed by these excellent reviews (61, 62), mainly due to the deregulation of methionine synthesis and salvage pathways. In addition, a metabolic cell cycle checkpoint related to methionine metabolism-controlled SAM/SAH (S-adenosylhomocysteine) ratio is crucial for tumor cell survival when undergoing methionine-deprived conditions (63). Recently, oncogenic mutation of PI3KCA has been shown to divert homocysteine into trans-sulfuration pathway, resulting in less generation of methionine from homocysteine and promoting methionine dependency in breast cancer cells (64). While the detailed mechanisms by which methionine dependency is formulated in tumor cells remain unclear, the efforts of utilizing this vulnerability in cancer therapy are already devoted for many years. Dietary methionine restriction has shown significant anti-tumor effects in pre-clinical animal models and no obvious side-effects in Phase I human clinical trials (65–68). Besides, methioninase, a methionine depleting enzyme, is an alternative way to deplete extracellular methionine source of tumor, which has been tested to successively suppress tumor growth (69–71). In addition, methioninase exhibits synergistic efficacy against tumors *in vitro* and *in vivo* when administered in combination with chemotherapy (72–74), highlighting its potency of enhancing first-line therapy in cancer treatment.

### Cysteine

Cysteine is one of the few sulfur-containing amino acids, which is mainly derived from the reduction of cystine (the oxidized form of two cysteines) imported from extracellular environment or transsulfuration pathway (generating cysteine from methionine metabolism). In addition to its proteogenic role, cysteine is a rate-limiting factor in the synthesis of glutathione (a tripeptide comprised of cysteine, glutamic acid and glycine), which is crucial for redox homeostasis (75). Cancer cells generally have high levels of metabolic turnover which easily results in accumulation of reactive metabolites, such as ROS (76). While elevated oxidative stress on one hand promotes oncogenesis below the lethal level, on the other hand, these reactive metabolites can covalently modify both proteins and DNA, eventually killing cells at high levels (77). Therefore, disruption of cysteine or glutathione metabolism which mediates the detoxification of toxic intermediates is presumed to have more adverse effects on cancer cells rather than normal cells. The major supply for intracellular cysteine is amino acid transporter SLC7A11/xCT-mediated cystine uptake and the subsequent reduction of cystine to cysteine (78). SLC7A11 has been found upregulated in multiple types of tumors and transcriptional inhibition of SLC7A11 is linked to tumor suppression controlled by tumor suppressor genes including p53, BAP1 and KEAP1 (79–82). Therefore, targeting SLC7A11-mediated cystine uptake to destroy antioxidative system in cancer cells with impaired glutathione synthesis or compromised transsulfuration pathway should be a feasible strategy to induce cystine auxotrophy (83, 84). Indeed, a chemical drug called erastin targeting SLC7A11 is identified and exhibits great lethality in human tumor cells through an oxidative cell death known as ferroptosis (85), which rapidly becomes a hot topic in the research field of regulated cell death (RCD). Systemic depletion of cystine through cyst(e)inase or genetically knockout of SLC7A11 significantly suppresses tumor growth in leukemia stem cells and genetically-engineered mouse models with chronic lymphocytic leukemia or pancreatic tumor (86–88). Thus, SLC7A11/xCT represents a novel therapeutic target for tumors that selectively experienced oxidative stress and exhibit a higher demand for antioxidants such as glutathione. This might also be determined by genetic status of other key players in reshaping cancer metabolism. For example, ARID1A (AT-rich interaction domain 1A)-deficient cancer cells, of which enhanced SLC7A11 expression by ARID1A-mediated chromatin remodeling disappears, are more susceptible to inhibition of the antioxidant glutathione due to excessive amounts of ROS-triggered apoptosis (89).

### Serine

Serine is another non-essential amino acid that causes dependency in a context-dependent manner, which can be taken up from extracellular resources or synthesized *de novo* from glycolysis intermediates or amino acids like glycine. Except for its proteogenic function, serine participates in several biosynthetic pathways including folate and methionine cycle through one-carbon metabolism, ultimately contributing to nucleotides synthesis, methylation reactions and redox buffering (90, 91). Serine auxotrophy has been discovered in rat myoblast line almost 50 years ago, possibly due to the limited biosynthetic capacity of these cells cultured *in vitro* (92). The first enzyme in the *de novo* serine synthesis pathway (SSP)—phosphoglycerate dehydrogenase (PHGDH) is found up-regulated in melanoma and breast cancers through genomic amplification by increasing the copy number of the gene, which adequately support cancer cell growth in the absence of serine (93, 94). The expression of another SSP enzyme phosphoserine aminotransferase (PSAT), which is downstream of PHGDH, also has a decisive role in terms of controlling serine dependency in breast tumors (95). Therefore, insufficient synthesis and increased demand of serine during tumor growth make the extracellular serine supply become a limiting factor that suppresses tumor development in various contexts. Serine starvation in p53-deficient tumor cells induces oxidative stress and reduces cell viability *in vitro* and *in vivo*, highlighting the potential role of targeting serine dependency in the treatment of tumors with p53 deficiency (96). Similarly, serine restriction sensitizes glioma cells to hypoxia-induced cell death through disrupting redox homeostasis (97). Besides, metabolic rewiring caused by genetic factors or pharmacologic intervention imposes tumor cells relying on exogenous serine to survive. For instance, oncogenic transcription factor EWS-FLI1 can impact Ewing sarcoma cellular metabolism and serine deprivation strongly inhibits Ewing sarcoma cell proliferation and tumorigenesis (98). Small molecule targeting PKM2 to activate glycolysis impedes serine synthesis pathway and induces serine auxotrophy in lung cancer cells (99). Practically, it still lacks an efficient way to deplete serine in anti-cancer treatment. The alternative choice is to use low-serine diet or PHGDH inhibitors, which appears promising in preclinical mouse models, yet remains to be exploited for therapeutic benefit in patients with cancer (90, 100, 101).

### Glucose

The critical role of glucose in supporting tumor growth has been widely studied since Otto Warburg discovered that cancer cells consume tremendous amounts of glucose for glycolysis even in the presence of oxygen in the 1920s (77, 102, 103). This phenomenon, also known as the Warburg effect, represents a striking metabolic characteristic that distinguishes tumors from normal tissues. Based on this difference, up-regulated glucose uptake by cancer cells has been successfully applied in diagnosis and evaluating response to treatment of patients with various types of solid tumors, through the use of fluorodeoxyglucose positron emission tomography (FDG-PET) imaging (104). Glucose metabolism contributes to tumor growth in multiple ways, including energy production, intermediated metabolites generation for the synthesis of nucleotides, amino acids and lipids as well as maintaining redox homeostasis (105). Targeting glucose metabolism, including downstream branches of glycolysis, pentose phosphate pathway and TCA cycle, has been extensively studied for several decades, and some of the drugs against key transporters or enzymes involved in glucose metabolism have been brought into clinical trials of cancer therapy, such as 2-Deoxy-D-glucose (2-DG) and metformin (105–108). Currently, two major features are considered as hall marks of glucose metabolism in tumor cells: increased glucose uptake and aerobic glycolysis. Oncogenic functions of genes such as Ras, cMyc, PI3K and LKB1 are found to elevate glucose uptake or up-regulate enzymes participating in glycolysis to promote tumorigenesis (109–112). Conversely, tumor suppressor genes like PTEN (phosphatase and tensin homolog) and p53 have the capacity to “cool down” glucose metabolism through inhibiting glycolysis or PPP (pentose phosphate pathway) (113, 114). Glucose limitation-caused redox imbalance has long been studied, largely due to the fact that PPP contributes to the most NADPH (nicotinamide adenine dinucleotide phosphate) generation in cytosol (115). Therefore, tumor cells undergoing high oxidative stress should be susceptible to glucose deprivation-mediated therapy. This is exactly the case in tumor cells with high SLC7A11/xCT expression, which is demonstrated to consume large amounts of NADPH during reduction of imported cystine in recent publication (116). Thus, SLC7A11-high tumors would be dependent on glucose/PPP-generated NADPH to prevent oxidative damage and more sensitive to glucose depletion or glucose transporters (GLUTs) inhibitor treatment (116, 117). Furthermore, changes of genetic background which leading SLC7A11 up-regulation such as KEAP1 mutation, can impose metabolic vulnerability of lung cancer cells to GLUTs inhibitors (82). These findings provide new insights when studying nutrient dependency, since the huge demand of one nutrient (such as cystine) may cause co-dependency of another nutrient (such as glucose) to maintain the metabolic balance inside cells (118).

### Lipids

Lipid metabolism within tumors is much more complicated considering the complex groups of biomolecules and a large number of forms for each subgroup that constituting lipids. Besides the indispensable role in cellular membrane construction, lipids also act as signaling molecules, provide energy sources and maintain redox homeostasis (119). There is no doubt that lipids are essential for cancer cell proliferation, and emerging evidence underlying their metabolic dysregulation have prompted new approaches toward cancer therapy (120–123). However, refined technologies including chromatography and mass spectrometry are required to differentiate specific lipid, resulting in fewer perspectives developed on lipid dependency from studies *in vitro*. Here we discuss two examples to give an intriguing idea about the crucial regulation of lipids within tumor cells. Sphingolipid metabolism is broadly reviewed in previous publications (124, 125), highlighting its use as promising target in cancer therapy. Indeed, preclinical use of acid sphingomyelinase which cleaves the sphingolipid or sphingomyelin into ceramide has been demonstrated in cancer therapy (126). In addition, molecules such as fenretinide, safingol, ABC294640, ceramide nanoliposomes (CNLs), SKI-II, α-galactosylceramide, fingolimod and sonepcizumab that modulating sphingolipid signaling have been exploited to induce cancer cell death through apoptosis or autophagy dependent manners (127). However, it remains unclear to what extent cancer cells exhibit dependency on extracellular sphingolipids, since sphingolipids imported from fetal calf serum *in vitro* are entirely catabolized by cultured cells and the role of sphingolipid-transporting proteins as cancer therapeutic targets remains elusive (128, 129). Cholesterol dependence is originally described in NS0, a nonsecreting mouse myeloma cell used for recombinant antibody production and dependent on an exogenous supply of cholesterol for survival and growth (130, 131). Increasing evidence demonstrate cholesterol metabolism and auxotrophy as targetable vulnerability in several cancers including pancreatic adenocarcinoma, glioblastoma, lymphoma and clear cell renal carcinoma, while key proteins facilitating cholesterol uptake like low-density lipoprotein receptor (LDLR), liver X receptor (LXR) and scavenger Receptor B1 (SCARB1) serve as ideal druggable targets to disrupt cholesterol metabolism (132–137). Additional choices could be to target *de novo* cholesterol synthesis enzymes or employ cholesterol lowering reagents like statins, which involve the complex signaling pathways in the regulation of cholesterol biosynthesis (138, 139).

### Vitamins

Vitamins are a group of organic compounds present in minute amounts within natural foods and important for biological functions including protein and energy metabolism, nutrient digestion, building blocks and redox balancing. Like amino acids mentioned above, increasing demands and/or deregulated expression of transporters are prone to generate vitamin dependence. Down-regulation of thiamine (also known as vitamin B1) transporter SLC19A3 in breast tumors presents a nutritional vulnerability and imposes cancer cells susceptible to acute thiamine starvation caused by thiaminase I enzyme treatment (140–143). However, thiamine deficiency is possibly linked to delirium (reduced mental abilities in thinking and sensing the environment) in cancer patients according to a retrospective descriptive study, suggesting a potential damage to the brain health caused by thiamine deprivation (144). A CRISPR/Cas9 functional genomic screen targeting metabolic enzymes found that pyridoxal kinase (PDXK, an enzyme that produces pyridoxal phosphate (PLP) from vitamin B6) acts an acute myeloid leukemia (AML)-selective dependency (145). However, the effects of vitamin B6 on tumor progression and therapeutic responses seemed controversial in previous reports, since high expression of PDXK has been implicated to constitute a good prognostic marker in patients with NSCLC (146). The distinguished effects of vitamin B6 metabolism could be at least partially explained by the different cell types, because depletion of vitamin B6 in culture media suppressed the proliferation of AML cells but not that of fibroblasts. Pharmacological inhibition of the vitamin B6 pathway significantly suppressed proliferation of leukemia cells and improved survival in mice, signifying the great potential of targeting vitamin B6 metabolism in anti-leukemia treatment (145). Further studies are needed to assess whether other molecules targeting vitamin B6 metabolism would have similar anti-cancer effects or not, like PDXK inhibitor artemisinins (147).

Recently, a systematic survey of nutrient dependencies has been performed t to identify genetic dependencies needed for the growth of AML cells *in vivo*, and myo-inositol transporter SLC5A3 was identified as a unique dependency to AML (148). Myo-inositol is not an essential nutrient considering it can be synthesized from glucose 6-phosphate through several enzymes like ISYNA1 and IMPA1, but myo-inositol was once considered to belong to the vitamin B family (148). Recurrent transcriptional silencing of ISYNA1 might largely contribute to the SLC5A3-mediated myo-inositol dependency in AML patients, since gain- and loss-of-function experiments were employed to unveil a synthetic lethal genetic interaction between ISYNA1 and SLC5A3 (148), indicating that combined treatments with SLC5A3 and ISYNA1 inhibition together could be exploited in AML.

### Possible Mechanisms Causing Nutrient Dependency

**Figure 2:**
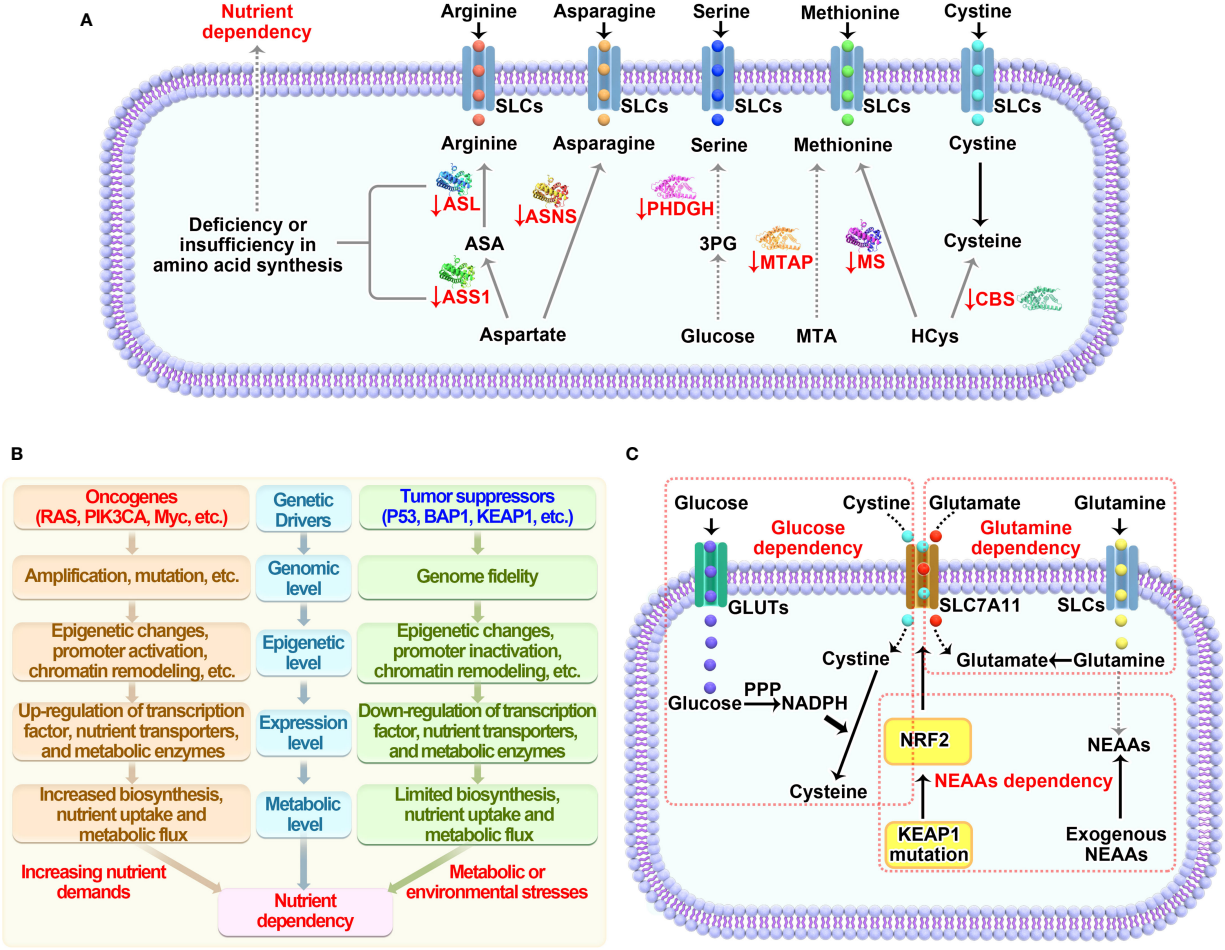
Mechanisms underlying nutrient dependency. **(A)** Deficiency or insufficiency in amino acid synthesis within cells causes dependency on extracellular nutrients. **(B)** Genetic factors including oncogenes and tumor suppressors either directly regulate the expression of transporters and enzymes mediating nutrient metabolism or indirectly control the demands needed for cell growth, which imposing specific dependencies on certain nutrients. **(C)** Nutrients involving crosstalk in their metabolic pathways are prone to be co-dependent on each other to maintain cellular homeostasis. SLCs, solute carrier-type transporters; GLUTs, glucose transporters; ASS1, argininosuccinate Synthase 1; ASL, argininosuccinate lyase; ASNS, asparagine synthetase; MTAP, methylthioadenosine phosphorylase; MS, methionine synthase; CBS, cystathionine beta-synthase; PPP, pentose phosphate pathway; HCys, homocysteine; MTA, S-methyl-5’-thioadenosine; NADPH, reduced nicotinamide adenine dinucleotide phosphate; NEAAs, non-essential amino acids.

Based on the discussions above, we are trying to summarize the common reasons that introduce specific nutrient dependency, aiming to help improve our current understanding of the regulation of such metabolic vulnerability in tumor cells (**Figure 2**).

### Deficiency or Insufficiency in *De Novo* or Salvage Synthesis

Generally, the capacity of synthesizing amino acids directly determines the extent of dependency on specific amino acid imported from extracellular nutrient pools. Down-regulation or even loss of ASS1 and ASL that required for arginine synthesis from aspartate causes arginine-dependency (13–16); The expression of ASNS controls *de novo* asparagine synthesis and modulates tumor cell sensitivity to ASNase treatment mediated asparagine depletion (28, 29). Low levels of methionine synthase (MS) or deletion of methylthioadenosine phosphorylase (MTAP) which salvage methionine through polyamine metabolism could explain methionine dependence (62, 149). Blocking serine synthesis pathway through silencing PSAT causes serine auxotrophy in luminal breast tumors (95). By contrast, functional supply of nutrients from *de novo* synthesis readily supports tumor growth under limiting conditions. Lineage-specific expression of glutamine synthetase (GS) makes luminal-type breast cells less glutamine-dependent compared with basal-type breast cells, which implies the ability of GS in predicting glutamine metabolism and dependency among breast tumor subtypes (45). The transsulfuration pathway that generating cysteine through methionine metabolism is favorable to tumor cell growth in the absence of extracellular cyst(e)ine (150). Alterations of the expression of rate-limiting enzymes involved in *de novo* or salvage synthesis for these nutrients represent a basic mechanism to cause tumor likely dependent on respective nutrient (**Figure 2A**). Therefore, the fundamental mechanisms underlying dysregulation of these enzymes in different tumor contexts likely lead to new options for targeting nutrient dependency, yet to be investigated in the future.

### Genetic Alteration-Induced Metabolic Reprogramming

Common genetic alterations including gene amplification, mutation and deletion in genes that play a central role in regulating gene expression and growth factor signaling cascade are able to drive specific metabolic shifts (**Figure 2B**). Such changes benefit cancer cells by enabling them to generate metabolic ingredients needed for supporting biomass synthesis as well as for adapting fluctuated stress environment. The oncogenic functions of RAS and Myc have been linked to nutrient fluxes regulation of glucose, glutamine and amino acids (110, 111, 151, 152). Active form of RAS (G12V) causes suppression of mitochondria function and elevated glycolysis to enhance tumor development *in vivo* (110). Upregulation of Myc promotes expression of genes involved in glutamine metabolism such as glutaminase and leads to glutamine addition (153, 154). Activation of PI3K/AKT pathway triggered by growth factor stimulation, oncogenic mutation of PIK3CA (encoding the p110α catalytic subunit of PI3K and/or loss-of-function mutations and deletions in PTEN (a negative regulator of PI3K signaling), is known to coordinate multiple metabolic programs for supporting tumor cell growth and proliferation (112). Particularly, AKT-mediated up-regulation of GLUT1 and GLUT4 directly promotes glucose uptake (155). Oncogenic mutation of PI3KCA reroutes metabolite from methionine cycle to trans-sulfuration pathway in cysteine metabolism which consequently causes methionine dependency (64 Loss of tumor suppressors could potentially mitigate nutrients dependency and make cancer cells survive under extreme conditions. P53- or BAP1-deficiency in tumor cells increases SLC7A11 expression and decreases cystine dependency, which is important for tumor growth *in vitro* and *in vivo* (79, 80). Of note, loss of tumor suppressor KEAP1 in lung cancers leads to glucose dependency through upregulating cystine metabolism-mediated NADPH consumption (82), suggesting an emerged metabolic vulnerability due to genetic alteration of such gene. While concurrent mutations of oncogenes and tumor suppressors are widely distributed according to cancer genomic studies, a broader insight into metabolic reprogramming in such context is necessary. KRAS/LKB1 co-mutant tumors have a higher activation of the hexosamine biosynthesis pathway (HBP), making them more dependent on the HBP enzyme glutamine-fructose-6-phosphate transaminase 2 (GFPT2) and defining a new metabolic vulnerability in such types of cancers (156).

### Metabolic Co-Dependency on Different Nutrients

The complexity of the metabolic pathways and the interactive functions of intermediate metabolites are likely far way ahead of our understandings and always in a dynamic change based on the specific genetic and/or biochemical contexts with differing nutrients availability. Many nutrients have redundant roles in regulating essential biological functions such as amino acids synthesis and redox homeostasis (42, 77, 117, 157). Thus, limiting one nutrient can lead to dependency on alternative nutrients for tumor cells to survive (**Figure 2C**). Glutamate is a crucial nitrogen donor for transamination reactions that promoting the synthesis of non-essential amino acids (NEAAs), while depletion of intracellular glutamate level by genetic mutation of KEAP1 or pharmacological inhibition of glutaminase promotes dependency on exogenous supply of NEAAs (158). This suggests an effective therapy for lung cancer patients with wide-type KEAP1 through combined treatment of glutaminase inhibition and NEAA deprivation. Surprisingly, high demand or utilization of one nutrient might also generate a druggable dependency on exogenous nutrients. SLC7A11, as we described early, imports a key amino acid cystine that is required for providing cysteine and maintaining redox balance through GSH synthesis (78). However, recent studies, including ours, have discovered that high expression of SLC7A11 mediated cystine uptake causes elevated dependency on exogenous glucose (117, 159). Further investigations confirm that reduction of cystine to cysteine consumes large amounts of NADPH generated through PPP, which is substantially inhibited when glucose is removed or glucose transporters are inhibited by GLUTs inhibitor (116). It appears that SLC7A11 acts as a double-edged sword in cellular redox regulation, making SLC7A11 an ideal metabolic target. Theoretically, you can always find a way to fight against cancer based on SLC7A11 expression level: restricting cystine or methionine in SLC7A11-low cells, or withdrawal of glucose in SLC7A11-high cells.

### Therapeutic Application of Targeting Nutrient Dependency

**Figure 3:**
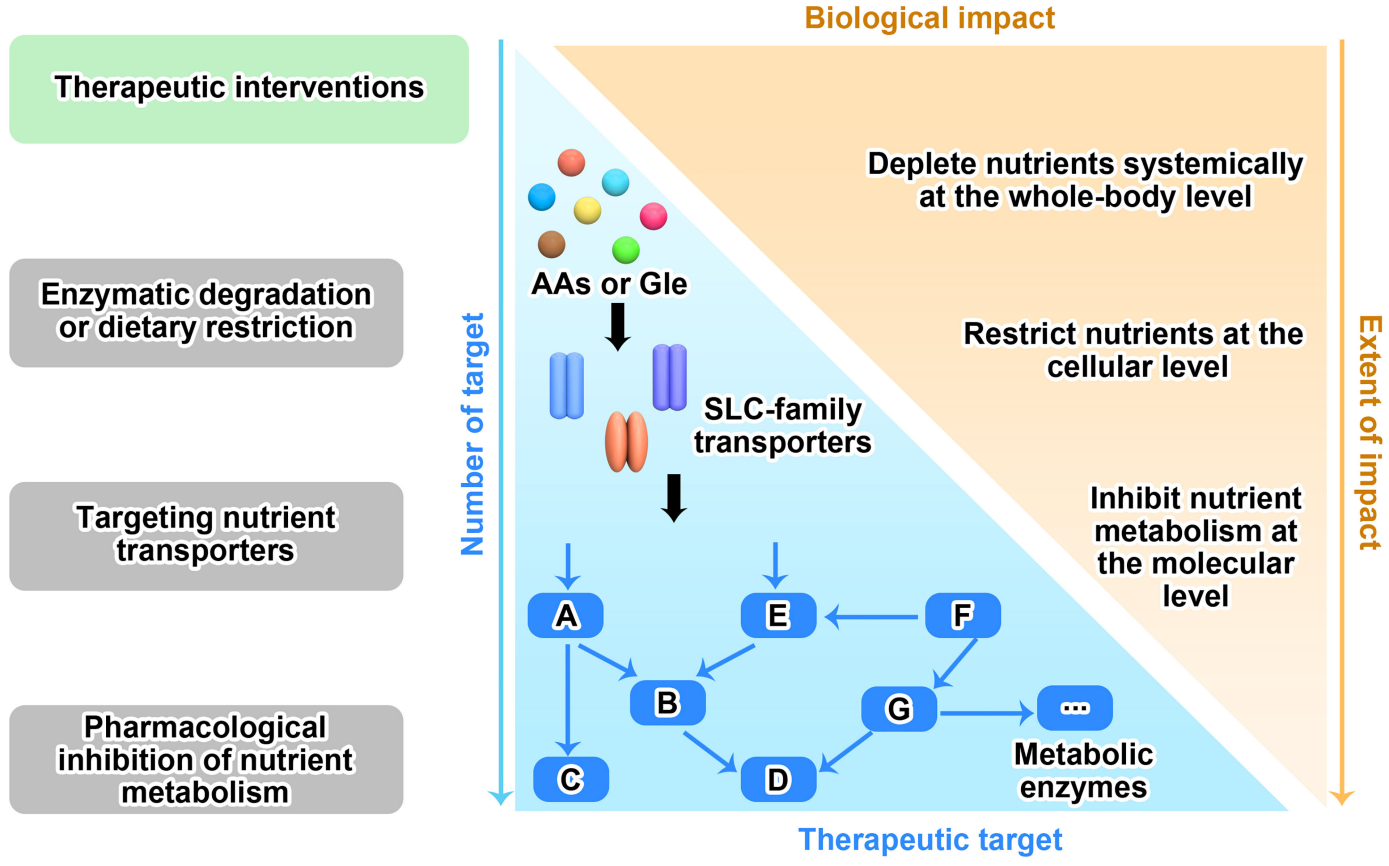
Therapeutic strategies for targeting nutrient dependency. Based on the nutrient availability in surrounding environment, nutrient transport on cell membrane and nutrient metabolism within cell, three therapeutic interventions that targeting nutrient dependency are wildly explored in the fields. Like the two sides of a coin, there are pros and cons for each strategy as well. The more therapeutic targets that clearly identified in the metabolic network, the more chance we are able to translate this strategy into clinical application. However, blocking any one of these targets may only have moderate biological functions *in vivo*, since each target has very limited functions compared to the whole metabolic network.

Therapeutic interventions through targeting nutrient dependency show great promise in the treatment of cancer. There are three rational ways to do this: 1) deplete nutrients in the extracellular context; 2) block transportation and suppress uptake of nutrients; 3) inhibit nutrient-derived metabolism. Accordingly, the below strategies have been developed to achieve the goal (**Figure 3**).

### Enzymatic Degradation- or Dietary Restriction-Mediated Nutrient Depletion

As described above, many nutrients depleting enzymes are demonstrated to be capable of degrading individual amino acid *in vitro* and *in vivo*. Asparaginase (ASNase), arginase (ARGase), arginine deiminase (ADI), methioninase and cyst(e)inase are successfully developed enzymatic drugs targeting their individual amino acids including asparagine, arginine, methionine and cyst(e)inase (6, 7, 9, 160). Compared to traditional chemical agents, the major advantage is their limited or controllable toxicity, considering their existence and physiological functions within human body. While bacterial-derived asparaginase has been approved to treat acute lymphoblastic leukemia and non-Hodgkin lymphoma, the therapeutic efficacies of other amino acids- depleting enzymes in multiple types of cancers are still under clinical investigation (9, 161). However, there are additional concerns that potentially undermine their clinical evaluation. If using therapeutic enzymes of non-human origin, allergic reactions due to the immunogenicity of the enzymes might preclude the continuous treatment if serious side effects such as anaphylactic shock arise (162). Besides, despite the relatively high specificity of amino acid depleting enzymes, they might have dual-enzyme activities and target secondary amino acid or substrate, particularly when the level of prime target is low. ASNase, which mainly catalyzes the hydrolysis of asparagine, also exhibits glutaminase activity that generates glutamate from glutamine, which is not required for its anti-cancer capability and even induces cytotoxicity in leukemia cells (33, 34). In addition, like all other types of drugs, the stability and penetrance preclude to widely apply amino acids-depleting enzymes in solid tumors that are usually surrounded by stroma and immune cells as well as connecting tissues. To overcome these challenges, new strategies such as chemical modification and engineered-biomaterials are employed to boost the efficacy of therapeutic enzymes. Pegylated enzymes that are covalently linked to polyethylene glycol (PEG), including PEG-asparaginase, PEG-arginase, PEG-arginine deiminase and PEG-methioninase, have been developed to decrease the immunogenicity and prolong the half-life (19, 23, 163, 164). Erythrocyte-encapsulated asparaginase (eryaspase) has less toxicity and improves patient survival in clinical trials for the treatment of patients with pancreatic cancer or acute lymphocytic leukemia when combined with chemotherapy (165, 166). These findings shed light on the translation of amino acids-depletion enzymes into clinical application in cancer treatment.

In contrast to dietary supplementation of specific nutrients, dietary restriction of individual nutrient is not as feasible and effective as we expected in the clinical study, considering the complex components in the food we digest and each patient’s compliance in the real world. For now, only dietary methionine restriction has moved into clinical trials, in which tested patients just showed modest decreases in plasma methionine levels (66). However, methionine-free diet combined with chemotherapy such as 5-fluorouracil and mitomycin C caused better responses in patients with gastric cancer compared with control treatment (methionine supplementation combined with the same chemotherapy) (167), while the underlying mechanisms remain unclear. Dietary restriction of glucose is almost impossible and usually achieved by alternative ways such as fasting and ketogenic diets (which divert energy intake from carbohydrate sources to fat). However, they might not be able to reduce glucose level efficiently at the systemic level (168). In this case (169), the exact role of dietary restriction like fasting in cancer-protection is not simply due to modulation of glucose dependency, which involves additional responses including T cell-dependent response and insulin signaling (170). Therefore, additional approaches to target nutrient dependency should be explored further in clinical trials, alone or in combination with other therapies.

### Targeting Nutrient Transporters

Cells rely on transporter proteins in the plasma membrane to acquire substrates such as amino acids and glucose. These transporters belong to a large family known as solute carrier (SLC) transporters that contain more than 300 different members and mediate fundamental physiological functions (171–173). Given the broad substrate specificity of most transporters, it leaves us little choice to choose the appropriate target that regulates nutrient dependency. SLC7A11/xCT is such a target we can utilize in nutrient transporter inhibition-mediated tumor therapy. Several molecules have been demonstrated to be capable of suppressing cystine uptake promoted by SLC7A11, such as erastin, sulfasalazine and sorafenib (174, 175). But the major issue for these compounds remains the same: specificity. Initially, erastin was found to target voltage-dependent anion channels (VDACs) and induce non-apoptotic cell death, later named as ferroptosis (85, 176). Sulfasalazine has been approved for medical use in the treatment of rheumatoid arthritis, ulcerative colitis, and Crohn’s disease several decades ago, yet the underlying mechanisms are still elusive (177). The incidental findings that sulfasalazine inhibits SLC7A11 make it attract more attentions in the field of ferroptotic cell death study (85, 178), which is a classic example of new uses for an old drug. Similarly, sorafenib is a multikinase inhibitor, primarily targeting both Raf and VEGF and PDGF receptor tyrosine kinase signaling (179). Glucose transporter inhibitors (GLUTi) have long been of great interest in the therapeutic study of targeting tumor metabolism, along with the discovery of multiple potent GLUTi (180). Though these drugs show clear inhibitive effects on glucose transporting activity, in most cases, they fail to suppress tumor growth *in vivo*, including the first highly GLUT-1 selective compound BAY-876 (181). There are many factors that affect the efficacy of a particular drug *in vivo*, such as the origin of tissue, tumor environment and genetics. Recent studies suggest high SLC7A11 expression promotes glucose dependency, which could serve as a biomarker for using GLUTi in cancer treatment (116, 117, 159). Thus, in addition to the generation of extremely specific molecules targeting individual nutrient transporter, context-dependent mechanisms underlying the efficacy of each molecule should also be extensively studied to guarantee its clinical translation.

### Pharmacological Inhibition of Nutrient Metabolism

Once entering into cells, nutrients undergo various metabolic pathways to meet the great demand for tumor growth or maintaining intracellular homeostasis. Thus, the rational intervention strategy would be targeting metabolic enzymes involved in these pathways. Glutaminase, the key enzyme responsible for the conversion of glutamine to glutamate, is considered to be a valuable therapeutic target for modulating glutamine/glutamate dependency. Small molecule CB-839 is one of the few glutaminase inhibitors currently evaluated in clinical trials (182).IPN60090 is a glutaminase-1 selective inhibitor with exciting physicochemical properties in phase 1 clinical trials (183). PHDGH inhibitors such as BI-4916 and BI-4924 that aim to block serine biosynthesis have been reported for many years, while none of them have yet entered into clinical stage (184), calling for further improvement and modifications of candidate inhibitors through pharmacological and biomaterial engineering research. Glucose metabolism plays a dominant role in regulating cellular functions, of which multiple potential targets for cancer therapy are exploited in drug development. Hexokinase (HK), phosphofructokinase (PFK) and pyruvate kinase isozymes M1/M2 (PKM1/2) are ideal targets in glycolytic pathways (185). Glucose-6-phosphate dehydrogenase (G6PD), the first enzyme in the PPP pathway, is important for promoting redox homeostasis through generating NADPH and upregulated in many tumors (186). Besides, mutants of isocitrate dehydrogenase (IDH) involved in the TCA cycle that produce oncogenic metabolites contribute to tumorigenesis, which makes mutant IDH an ideal therapeutic target (187). Accordingly, small molecule inhibitors targeting these metabolic enzymes are consistently developed to take advantage of metabolic vulnerability within cancers (77). To give a better idea about the therapeutic translation of targeting nutrient dependency in cancer treatment, we summarize the most relative information in **Table 1**.

**Table 1:** Therapeutic exploitation of targeting nutrient dependency in cancer treatment.

Nutrients	Therapeutic interventions			Translational exploration			Experimental cancer types	References
	Depleting enzymes	Transporter inhibitors (*Target*)	Metabolic inhibitors (*Target*)	Pre-clinical	Clinical	FDA approved*		
**Amino acids**								
Arginine	(PEG-) ARGase	NA	DFMO (ODC)	ARGase	ARGase (Phase III)	NA	Prostate cancer, Non-small cell lung cancer, Solid tumors, Glioma, Acute myeloid leukemia, Advanced pancreatic cancer, Malignancies, Colon cancer, Skin cancer, Glioblastoma, Breast cancer, Hepatocellular carcinoma, Melanoma, Glioblastoma multiforme, Pancreatic cancer, Lymphoma, Soft tissue sarcoma, Mesothelioma	(6, 7, 11, 16–19, 21–24, 160)
	(PEG-) ADI			ADI	ADI (Phase I/II)			
				DFMO				
Asparagine	(PEG)-ASNase	NA	APR-246 (ASNS)	NA	APR-246 (Phase I/II)	(PEG)-ASNase^a^, Eryaspase^b^	Adenocarcinoma, Glioblastoma, Glioma, Non-small cell lung carcinoma, Ovarian cancer, NK/T-cell lymphoma, T-cell lymphoma, Bladder cancer, Pancreatic cancer, Acute myeloid leukemia, Acute lymphoblastic leukemia, Triple-negative breast cancer, Non-Hodgkin lymphoma	(6, 7, 27–29, 33, 35, 37, 164–166, 185)
	Eryaspase							
Glutamine	Glutaminase	Benzylserine, γ-FBP, GPNA, V-9302 (SLC1A5)	CB-839, IPN60090, C968, BPTES (GLS)	Glutaminase	CB-839 (Phase I/II)	NA	Myeloma, Glioma, Head and neck squamous cell carcinoma, Non-small cell lung cancer, Breast cancer, Acute myeloid leukemia, Hepatocellular carcinoma, Lymphoma, Glioblastoma multiforme, Bladder cancer, Sarcoma, Triple-negative breast cancer, Ovarian cancer, Colon cancer, Colorectal cancer, Melanoma, Waldenstrom macroglobulinemia, Plasma cell myeloma, Astrocytoma, Acute lymphoblastic leukemia	(33, 34, 36, 50–55, 182, 183)
			EGCG, R162 (GLUD)	Benzylserine, γ-FBP, GPNA, V-9302	IPN60090 (Phase I)			
			AOA (Aminotransferase)	C968, BPTES				
				EGCG, R162				
				AOA				
Methionine	(PEG-) Methioninase	NA	FIDAS-5, PF-9366, AG-270 (MAT2A)	FIDAS-5	AG-270 (Phase I)	NA	Colon cancer, Breast cancer, Neuroblastoma, Lung cancer, Renal cancer, Lymphoma, Prostate cancer	(58, 60, 64, 70–74, 110)
				PF-9366	Methioninase (Phase I)			
	
Cysteine	Cyst(e)inase	Erastin, IKE, SSZ, Sorafenib, Lanperisone (SLC7A11)	BSO (GCL)	Cyst(e)inase, Erastin, IKE, SSZ,	BSO (Phase I)	Sorafenib^c^, SSZ^d^, Lanperisone^e^	Breast cancer, Prostate carcinoma, Chronic lymphocytic leukemia, Pancreatic cancer, Colorectal cancer, Head and neck squamous cell carcinoma, Hepatocellular carcinoma, RAS-mutant cancers, Non-small cell lung cancer	(78, 79, 83–88, 116, 150, 174, 175, 177, 179)
			ATA (CSE)	ATA				
			RSL3, ML162, ML120 (GPX4)	RSL3, ML162, ML120				
	
Serine	NA	NA	BI-4916, BI-4924, PHGDH-hit, CBR-5884, PH-755 (PHGDH)	BI-4916, BI-4924, PHGDH-hit, CBR-5884, PH-755	NA	NA	Triple-negative breast cancer, Non-small cell lung carcinoma, Melanoma, B-cell lymphoma, Colon cancer	(90, 95, 96, 99–101, 184)
**Glucose**								
Glucose	NA	BAY-876, Apigegnin, WZB117, STF-31 (GLUT1)	2-DG, Lonidamine, 3-BP (HK2)	BAY-876, STF-31, 3-BP, WZB117	Apigegnin, Lonidamine (Phase I/II), 3-BP (Phase I)	Ritonavir^f^	Multiple myeloma, Ovarian cancer, Kidney cancer, Renal cancer, Lung cancer, Malignant gliomas, Osteosarcoma, Non-small cell lung cancer, Breast cancer, Bladder carcinoma, Skin cancer	(105–108, 117, 180, 181, 185)
		Ritonavir (GLUT4)	Shikonin, Alkannin, Orlistat (PKM2)	Shikonin, Alkannin, Orlistat	2-DG (Phase II)			
		2, 5-AM (GLUT5)	DHEA, 6-Aminonicotinamide, RRx-001 (G6PD)	2, 5-AM, 6-Aminonicotinamide	DHEA (Phase II)			
		Phloretin (SGLT1/2)	3PO, PFK15 (PFKFB3)	3PO, PFK15, Phloretin	RRx-001 (Phase III)			
			KA (GAPDH)	KA				
**Lipids**								
Sphingolipids	Sphingmyelinase	NA	C8-CPPC (DES), CHC (CERT), NVP-231 (CERK), LCL521 (AC)	C8-CPPC, CHC, NVP-231, LCL521	Sonepcizumab (Phase II)	FTY720^g^	Prostate cancer, Breast cancer, Acute myeloid leukemia, Lung cancer, Head and neck cancer cells, Leukaemia, Neuroblastoma, Colon cancer, Bladder cancer, Melanoma, Non-small cell lung carcinoma, Esophageal tumor, Ovarian cancer, Hepatobiliary cancer, Glioblastoma	(121–127, 129)
			SK1-I, PF543 (SPHK 1), ABC294640 (SPHK 2)	SK1-I, PF543, ABC294640				
			Sonepcizumab (S1P)	Sphingmyelinase				
			FTY720, VPC03090 (S1PR 1), JTE013, AB1 (S1PR 2)	JTE013, AB1, VPC03090				
Cholesterol	NA	Ezetimibe (NPC1L1)	Statins :lovastatin, mevastatin, atorvastatin, uvastatin, rosuvastatin, pitavastatin, simvastatin, pravastatin, fluvastatin sodium (HMG-CoA)	YM-53601	TAK-475 (Phase III)	Ezetimibe^h^, Statins^i^	Colorectal cancer, Breast cancer, Lung cancer, Prostate cancer, Pancreatic cancer, Myelogenous leukemia	(130–139)
			YM-53601, TAK-475 (SQS)	R048-8071	Exemestane (Phase IV)	Terbinafine^j^		
			R048-8071 (OSC), Exemestane (ARO)		Avasimibe (Phase III)			
			Avasimibe (ACAT1)		Lonafarnib, Tipifarnib (Phase III)			
			Lonafarnib, Tipifarnib (farnesyltransferase and certain bisphosphonates)					
			Terbinane (SQE or OSC)					
**Vitamins**								
Thiamine	(PEG-) Thiaminase I	NA	Pyrithiamine, Oxythiamine, Amprolium (thiamine antagonists)	(PEG-) Thiaminase I	NA	NA	Lymphoid leukemia, Clear cell renal cell carcinoma, Breast cancer	(140–143)
				Pyrithiamine, Oxythiamine, Amprolium				
Pyridoxine	NA	NA	Artemisinin (PDXK)	NA	NA	Artemisinin^k^	Acute myeloid leukemia	(145, 147)

PEG-, Polyethylene glycol; ARGase, Arginase; ADI, Arginine deiminase; DFMO, Difluoromethylornithine; ODC, Ornithine decarboxylase; ASNase, Asparaginase; ASNS, Asparagine synthetase; γ-FBP, γ-Folate binding protein; GPNA, L-γ-glutamyl-p-nitroanilide; GLS, Glutaminase; GLUD, Glutamate dehydrogenase; AOA, Aminooxyacetate; MAT2A, Methionine adenosyltransferase 2A; IKE, Imidazole ketone erastin; SSZ, Sulfasalazine; BSO, L-buthionine sulfoximine; GCL, Glutamate cysteine ligase; ATA, Aurintricarboxylic acid; CSE, Cystathionine γ-Lyase; RSL 3, Ras-selective lethal small molecule 3; GPX4, Glutathione peroxidase 4; PHGDH, Phosphoglycerate dehydrogenase; GLUT, Glucose transporter; 2, 5-AM, 2, 5-Anhydro-D-maaitol; SGLT, Sodium-dependent glucose transporters; 2-DG, 2-Deoxy-D-glucose; 3-BP, 3-Bromopyruvate; HK2, Hexokinase 2; PKM2, Pyruvate kinase M 2; DHEA, Dehydroepiandrosterone; G6PD, Glucose-6-phosphate dehydrogenase; 3PO, 3-(3-pyridinyl)-1-(4-pyridinyl)-2-propen-1-one; PFK15, 1-(4-pyridinyl)-3-(2-quinolinyl)-2-propen-1-one; PFKFB, 6-phosphofructo-2-kinase/fructose-2,6-bisphosphatases; KA, Koningic acid; GAPDH, Glycerladehyde-3-phosphate dehydrogenase; C8-CPPC, C8-cyclopropenylceramide; DES, Dihydroceramide desaturase; CHC, 3-chloro-8β-hydroxycarapin-3,8-hemiacetal; CERT, Ceramide transfer protein; CERK, Ceramide kinase; AC, Acid ceramidase; SPHK, Sphingosine kinase; S1P, Sphingosine-1-phosphate; S1PR, S1P receptor; NPC1L1, NPC1 like intracellular cholesterol transporter 1; HMG-CoA, 3-hydroxy-3-methylglutaryl-coenzyme A; SQS, Squalene synthase inhibitor; OSC, 2, 3-Oxidosqualene cyclase; ARO, Steroidal aromatase; ACAT1, Acetyl-CoA acetyltransferase 1; SQE, Squalene epoxidase; PDXK, Pyridoxal kinase; FDA, U.S. Food and drug administration; *Note, listed drugs may not be approved to treat cancers.

a , FDA approved drug to treat acute lymphoblastic leukemia and lymphoblastic lymphoma;

b , FDA approved drug to treat acute lymphocytic leukemia;

c , FDA approved drug to treat hepatocellular carcinoma;

d , FDA approved for the treatment of ulcerative colitis and rheumatoid arthritis;

e , FDA-approved muscle relaxant;

f , Protease inhibitors for treatment of AIDS;

g , FDA approved drug for multiple sclerosis;

h , FDA approved agent to inhibit cholesterol absorption in the intestine;

i , FDA approved drugs to reduce the amount of low-density cholesterol;

j , FDA approved drug for the treatment of onychomycosis of the toenail or fingernail due to dermatophytes;

k , FDA approved drug to treat malarial.

NA, Not available.

## Challenges and Future Directions

Growing evidence keep refreshing our understanding of tumor metabolic regulation, which literally are influenced by surrounding environment, cancer genetics and lineage. Tumor microenvironment represents a complex context either directly or indirectly interacting with tumor metabolism in response to alteration of nutrient availability (10, 188). Thus, a clear picture drawing the interaction between tumor metabolism and environmental perturbation is likely required for successful translation of targeting nutrient dependency of cancers *in vivo*. Physiochemical property and target specificity of nutrient degrading enzymes and small molecule inhibitors discussed above should be the threshold for the successful translation of nutrient dependencies into clinical interventions. Chemical modification, biomaterials and nanoengineering-mediated drug delivery have been introduced to improve the drug stability and efficacy, including pegylated modification and nanoparticle-mediated capsulation, which definitely deserves more investigation (19, 20, 22, 23, 164, 189). Another challenge is the limited efficacy of drugs targeting nutrient dependency as a single agent. However, accumulating studies demonstrate synergistic effects when nutrient depleting therapy is combined with other first-line anti-cancer treatment, such as immune check point inhibition and chemotherapy (9, 24). Therefore, increasing understanding of metabolic regulation within tumor cells allows for rational design and validation of combination therapies. For example, glucose deprivation-induced inactivation of PRC1 (polycomb-repressive complex 1) promotes ER (endoplasmic reticulum) stress and cell death, leading to the strategic combination of PRC1 inhibitor and GLUTi treatment in cancer cells (190). In summary, a deep understanding of tumor metabolism and nutrient dependency is the premise to bring our battle against cancer to the final stage.

## Author Contributions

All authors listed have made a substantial, direct, and intellectual contribution to the work and approved it for publication.

## Funding

The authors are grateful for the funding supports from National Natural Science Foundation of China (8210113396 to YZ), China Postdoctoral Science Foundation (2020M681922 to QX), Health Commission of Zhejiang Province (JBZX-201901 to QX) and Science & Technology Department of Xinjiang Uygur Autonomous Region (2021D01B95 to ZL). YZ is a scholar supported by Top Young Talents Programme at Xi’an Jiaotong University.

## Conflict of Interest

The authors declare that the research was conducted in the absence of any commercial or financial relationships that could be construed as a potential conflict of interest.

## Publisher’s Note

All claims expressed in this article are solely those of the authors and do not necessarily represent those of their affiliated organizations, or those of the publisher, the editors and the reviewers. Any product that may be evaluated in this article, or claim that may be made by its manufacturer, is not guaranteed or endorsed by the publisher.
